# Shrewd AKT regulation to survive

**DOI:** 10.18632/oncoscience.16

**Published:** 2014-03-17

**Authors:** Moez Dawood, Gordon B. Mills, Zhiyong Ding

**Affiliations:** Department of Systems Biology, The University of Texas MD Anderson Cancer Center, Houston, TX; Department of Systems Biology, The University of Texas MD Anderson Cancer Center, Houston, TX

Metabolic stress, such as an insufficient supply of nutrients or oxygen owing to inadequate tumor neovascularization, metastasis, or therapy, is a microenvironmental condition frequently encountered by cancer cells [1]. The ability to survive metabolic stress is thus a necessity for tumor initiation and progression. The serine/threonine kinase AKT (also known as protein kinase B) is a primary mediator of survival of both normal and malignant cells [2]. Our recent studies revealed that AKT could be differentially activated by site-specific phosphorylation according to the severity and duration of metabolic stress, enabling cells to shift gears to survive [3].

During canonical AKT activation, growth factors or other stimuli activate transmembrane receptor tyrosine kinases, which in turn activate phosphoinositide 3-kinase to phosphorylate phosphatidylinositol 4, 5-bisphosphate to form phosphatidylinositol 3, 4, 5-trisphosphate on the inner cell membrane. AKT and its upstream kinase, phosphoinositide-dependent kinase-1 (PDK1), are recruited to the cell membrane, which initiates AKT phosphorylation at Thr308 by PDK1 [4]. Mammalian target of rapamycin complex 2 and other potential PDK2 kinases phosphorylate AKT at Ser473, resulting in optimal AKT activation [5]. The active phosphorylated AKT then translocates from the cell membrane to other cell compartments to phosphorylate multiple downstream substrates to fulfill its versatile functions.

In contrast with canonical AKT activation with coordinate phosphorylation on both Thr308 and Ser473, we identified a novel AKT activation mechanism induced by glucose deprivation by which AKT is selectively phosphorylated on Thr308 but not Ser473, resulting in targeting AKT to a specific group of substrates [3]. In HeLa cells, short-term glucose deprivation (for up to 6 h) induced a modest increase in AKT phosphorylation at both Thr308 and Ser473. In contrast, prolonged glucose deprivation (16 h) induced a marked increase in AKT phosphorylation at Thr308 (up to 30-fold) but only a modest increase at Ser473 (twofold to threefold). Phosphorylation at Thr308 continued to increase over a 16-h glucose deprivation period, whereas Ser473 phosphorylation peaked at about 6 h and subsequently declined (Figure). Apparently, at least two independent processes are responsible for AKT phosphorylation during 16-h glucose deprivation, with a switch occurring between 6 and 8 h. Our data indicated that the first process takes place primarily via the release of feedback inhibition from p70S6K that results in coordinated phosphorylation of AKT at both Thr308 and Ser473. The second process likely occurs via the formation of a complex including GRP78, PDK1, and AKT that promotes selective AKT phosphorylation at Thr308.

**Figure:**
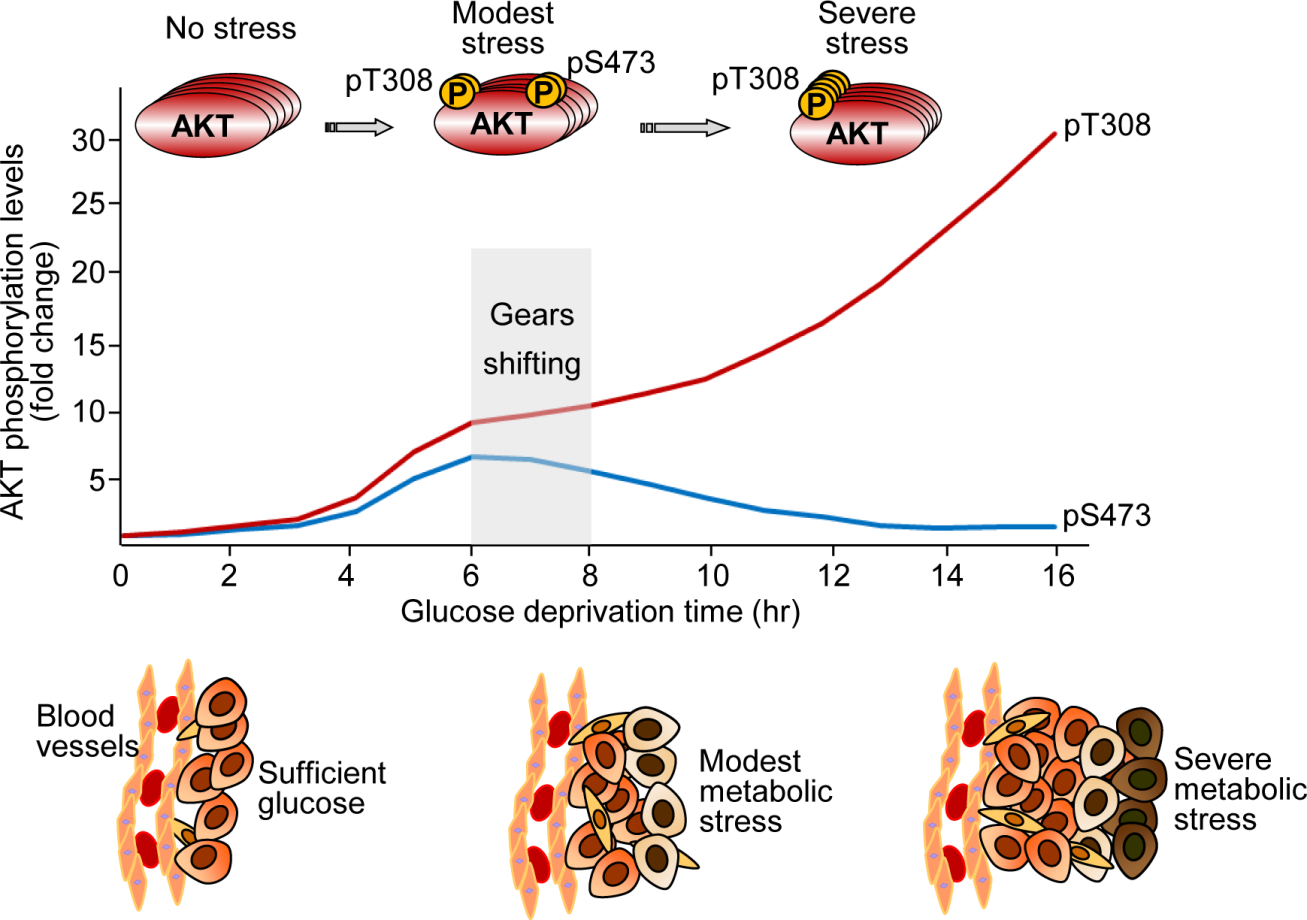
AKT phosphorylation on Thr308 and Ser473 induced by glucose deprivation and a proposed in vivo metabolic stress model Curves of AKT phosphorylation at Thr308 (pT308) and Ser473 (pT473) were generated from previously published data [3]. The shaded area (“Gears shifting”) indicates a proposed stage at which distinct AKT activation mechanisms switch. This proposed *in vivo* metabolic stress model is adapted from a previous publication with modifications [3].

AKT is functionally activated by selective Thr308 phosphorylation during prolonged glucose deprivation as indicated by increased phosphorylation of several known AKT substrates, including glycogen synthase kinase 3, mammalian target of rapamycin, and Y-box binding protein 1. Strikingly, several well-established AKT substrates, including PRAS40 and BAD, were not targeted by AKT under glucose deprivation conditions. Thus, with selective Thr308 phosphorylation, AKT may have altered substrate selectivity. We also observed coordinate phosphorylation of multiple residues other than Thr308 and Ser473 on single AKT molecules in an isoform-specific manner [6], which may also contribute to substrate selectivity. Therefore, AKT could be shrewdly activated for the right substrate spectrum under different cellular contexts, enabling cells to specifically survive energy crisis during metabolic stress.

Shrewd AKT activation may occur *in vivo*. When a tumor grows, some tumor cells located away from blood vessels may be subjected to glucose deprivation (Figure). We propose that these cells employ a layered defense against cell death according to the severity and duration of metabolic stress. Mild or transient metabolic stress (e.g., transient glucose deprivation) induces modest AKT phosphorylation at both Thr308 and Ser473, which provides the first line of defense against cell death. However, during prolonged glucose deprivation, this line of defense becomes insufficient to protect cells against death and is shut off. The next tier of the survival mechanism is then activated. It selectively and markedly increases AKT phosphorylation at Thr308, providing a second line of defense against cell death caused by severe metabolic stress. Therefore, cells are capable of shifting gears to survive specific harsh conditions. AKT, with its versatile activation mechanisms, provides a set of important survival gears that cells may use under specific conditions.

In summary, glucose deprivation induces site- specific phosphorylation and substrate-specific activation of AKT via distinct mechanisms according to the duration of metabolic stress. These mechanisms may explain how cells tolerate metabolic stress in tumors by using AKT as a survival tool. Our findings revealed a novel AKT-mediated survival mechanism under prolonged metabolic stress that is important to the development and implementation of drugs targeting cell metabolism and AKT signaling.
